# Epidemiology, antimicrobial resistance, and risk factors of infection among liver transplant patients: a retrospective study 2010–2023

**DOI:** 10.1128/spectrum.00711-25

**Published:** 2025-06-10

**Authors:** Lei Liu, Zhongyi Jiang, Huanjin Liao, Zhiwei Xu, Yang Yu, Lin Zhong, Pusen Wang

**Affiliations:** 1 Department of General Surgery, Shanghai General Hospital of Nanjing Medical University 12461, Shanghai, China; 2 Department of Liver Surgery and Organ Transplantation Center, Shenzhen Third People’s Hospital, Second Affiliated Hospital, Southern University of Science and Technology 535206 , Shenzhen, Guangdong, China; 3 Department of Laboratory Medicine, Shanghai General Hospital, Shanghai Jiao Tong University School of Medicine 56694, Shanghai, China; Central Texas Veterans Health Care System, Temple, Texas, USA

**Keywords:** **l**iver transplantation, pathogen, spectrum, resistance, sepsis

## Abstract

Liver transplantation (LT) recipients exhibit heightened susceptibility to infection and sepsis, which have emerged as the most prevalent and life-threatening complications significantly impacting prognosis. The etiological spectrum of organisms following LT has undergone substantial changes over recent decades. This retrospective monocentric study included a consecutive cohort of 776 LT patients from 2010 to 2023. A total of 207 pathogens were isolated from 180 infection sites of 156 (20.1%) patients, comprising 82 (39.6%) gram-positive bacteria, 90 (43.5%) gram-negative bacteria, and 35 (16.9%) fungi. Among the gram-negative bacteria, carbapenem-resistant *Enterobacteriaceae* (CRE) was found in eight cases (seven *Klebsiella pneumoniae* and one *Escherichia coli*), extended-spectrum beta-lactamases (ESBLs)-producing bacteria were detected in seven cases (five *Escherichia coli* and two *Enterobacter cloacae*), and carbapenem-resistant *Acinetobacter baumannii* (CRAB) was found in 14 cases and 2 cases had carbapenem-resistant *Pseudomonas aeruginosa* (CRPA). Advanced age, prolonged mechanical ventilation, and extended ICU stay were significantly associated with increased susceptibility to post-LT infections. Infected patients with bilirubin levels exceeding 90 µmol/L (OR 3.46, 95% CI 1.46–8.24; *P* = 0.005) as well as drug-resistant bacterial infections (OR 2.35, 95% CI 1.07–5.15; *P* = 0.033) were more likely to develop sepsis. More than 45% of bacterial infections were caused by drug-resistant pathogens, with over 30% of gram-negative bacteria exhibiting carbapenem resistance. In conclusion, the implementation of strategies aimed at reducing the duration of mechanical ventilation and ICU stay may effectively decrease the incidence of post-liver transplantation infection. Furthermore, pre-transplant interventions targeting reduction in jaundice could potentially mitigate the risk of post-transplant sepsis.

**IMPORTANCE:**

This study elucidates the recent pathogen spectrum associated with infections in liver transplantation and compares it with the pathogen spectrum from previous periods. Additionally, it analyzes high-risk factors for post-transplant infections and innovatively examines the risk factors that contribute to the progression from infection to severe infection and sepsis. Based on these findings, potential intervention strategies targeting infection and sepsis are proposed.

## INTRODUCTION

Liver transplantation (LT) has emerged as the best therapeutic approach for end-stage liver disease since 1963 (1). With significant advancements in LT surgical techniques, anti-rejection therapies, and organ preservation methods, perioperative infection has become a major challenge affecting recipients’ prognosis (2–4). Particularly during the period of donation after cardiac death (DCD) in China, the incidence and severity of infections are considerably higher due to donor ischemia-reperfusion injury and donor-derived infections. Furthermore, recipients are highly susceptible to infections due to prolonged administration of potent immunosuppressive agents. Studies have reported that infection occurs in 40%–89% of recipients within one year post-LT (5, 6).

Infection following LT is frequently caused by multi-drug resistant (MDR) bacteria, such as methicillin-resistant *Staphylococcus aureus* (MRSA), carbapenem-resistant *Enterobacteriaceae* (CRE), and others (3, 4). These infections often manifest as severe mixed infections involving fungi, bacteria, and viruses, which can progress to sepsis. Sepsis represents the most lethal infectious disease and contributes significantly to mortality rates in LT patients (7, 8). Kim et al. reported that bacteremia developed in 24% of LT recipients (9). According to the latest Sepsis-3 criteria, up to 67% of patients develop sepsis after LT (10). Previous studies have shown that LT patients have an approximately 15-fold increased risk of developing MRSA infection with a corresponding 30-day mortality rate of up to 21% (11, 12). Our own observations also revealed a strong association between MRSA infection and a one-month mortality rate of 41% (3). The mortality rate for carbapenem-resistant *Klebsiella pneumoniae* (CRKP) infection after LT has been reported as high as 33%–42% (13, 14).

Our previous study in 2012 demonstrated the spectrum and risk factors associated with MDR-negative bacilli infection in LT, which were the predominant causative pathogens leading to mortality at that time (4). However, there has been a shift in the infection spectrum after LT over these years, with an increased incidence of gram-positive bacilli and fungal infections. In 2022 and 2023, we also reported a high prevalence of MRSA infection and sepsis, providing further insights into the underlying molecular mechanisms (2, 3). Therefore, to investigate this evolving pattern of infections after LT, we conducted a large-scale retrospective study. It is well-recognized that therapeutic options for MDR bacteria and sepsis are extremely limited. The variation in infection patterns among different transplantation centers worldwide remains poorly understood. This study represents the first comprehensive analysis considering the infection pattern and associated risk factors of LT in East China, offering valuable guidance for clinical practice against infections and improving LT prognosis globally.

## MATERIALS AND METHODS

### Patients

Patients aged 18 years or older who underwent orthotopic LT between June 2010 and January 2023 were included in this study. Clinical data were obtained from the prospectively maintained database at Shanghai General Hospital. The exclusion criteria encompassed individuals who had undergone re-transplantation, combined organ transplantation, or ABO-incompatible transplantation. All LT procedures were performed by the same surgical team and managed in the intensive care unit (ICU) of Shanghai General Hospital. The immunosuppression protocol involves immune induction using basiliximab and methylprednisolone. For immune maintenance, we combine tacrolimus with mycophenolate mofetil. In cases of patients with renal insufficiency or tumors, we incorporate sirolimus into the regimen while simultaneously reducing the dose of tacrolimus.

### Definition of infection and sepsis

The occurrence of infection following LT was confirmed through culture or isolation, and subsequent resistance testing was conducted as previously described (4, 15). Evaluation of sepsis after LT was performed in accordance with the sepsis-3 criteria as previously outlined (2).

### Data collection

The recipient baseline and clinical data, including age, sex, etiology, diabetes, cirrhosis, albumin, creatinine, bilirubin, ascites, alpha-fetoprotein (AFP) levels, Child-Pugh grading, and the model for end-stage liver disease (MELD) score, were recorded. Additionally, the donor baseline and clinical data consisted of age, sex, albumin, bilirubin, aspartate transaminase (AST), alanine transaminase (ALT), as well as surgery characteristics such as warm ischemia time, cold ischemia time, time of anhepatic phase, time of operation, blood loss, time of mechanical ventilation, duration in ICU after LT, and infectious complications comprising peritonitis, pleural effusion, cholangitis, catheter-related sepsis, pulmonary infection, urinary infection, incision infection, and pathogens consisting of gram-positive bacteria, gram-negative bacteria, and fungus.

### Statistical analysis

The mean and standard deviation were used to express continuous data, while frequencies were used for discrete variables. Categorical variables were compared using Pearson’s χ^2^ test or Fisher’s exact test, whereas continuous variables were analyzed using Student’s *t*-test, the Mann-Whitney U test, or a one-way analysis of variance. Logistic regression models were employed to investigate independent risk factors for infection and sepsis. The final models included dichotomized variables with *P* values < 0.10 from univariate analysis in multivariate logistic regression analysis. All statistical analyses were performed using SPSS ver. 24.0 statistical software (SPSS Inc., Chicago, IL, USA). Statistical significance was defined as *P* < 0.05.

## RESULTS

### Recipient, donor, and surgery characteristics

A total of 776 patients who underwent LT were included in this study. The median age of the recipients was 47, with 83.4% being male. Hepatitis B virus (HBV) infection was the predominant etiology, accounting for 80.3%. Among the recipients, 11% had diabetes and 76.9% had cirrhosis. The mean levels of albumin, creatinine, and bilirubin were measured at 34.05 g/L, 79.02 µmol/L, and 89.81 µmol/L, respectively. Severe ascites was present in 21.4% of the recipients, while mild to moderate ascites was observed in 30.9%. The AFP level was found to be at a value of 622.6 ng/mL. Regarding disease severity classification, Child-Pugh grade A accounted for approximately 35%, grade B accounted for around 39%, and grade C represented about 25% of all recipients enrolled in this study. The average MELD score calculated among these patients was 14.43.

The median age of donors was 28, with 96.5% being male. The mean levels of albumin, bilirubin, AST, and ALT were 33.97 g/L, 17.02 µmol/L, 48.17 U/L, and 49.72 U/L, respectively. Regarding surgical characteristics, the average warm ischemia time and cold ischemia time were recorded as 3.56 min and 8.19 h, respectively. The mean duration of the anhepatic phase and the overall operation were found to be approximately 45.42 min and 6.62 h, respectively. The average blood loss during surgery amounted to approximately 1895 mL. Furthermore, the mechanical ventilation period post-LT averaged around 72.7 h while the ICU stay lasted for an average duration of 446.3 h. 1.7% of the patients received immune checkpoint inhibitors prior to LT, while 37.5% of the patients were able to discontinue prednisone treatment after LT. These specific characteristics are presented in Table S1.

### Infection sites, pathogens, and antibiotic resistance

Among the 776 enrolled patients, a total of 156 recipients were found to have acquired infections after LT. Infection sites were identified in a cumulative count of 180 cases, encompassing peritonitis (15 cases; 1.9%), cholangitis (10 cases; 1.3%), incision infections (19 cases; 2.4%), abdominal abscesses (5 cases; 0.6%), pulmonary infections (110 cases; 14.2%), infectious pleural effusion (7 cases; 0.95%), catheter-related sepsis (6 cases; 0.8%), and urinary tract infections (8 cases; 1%) as presented in Table 1.

**TABLE 1:** Summary of infection sites post-LT

Infection sites	*N* = 180
Peritonitis	15 (1.9%)
Cholangitis	10 (1.3%)
Incision infection	19 (2.4%)
Abdominal abscess	5 (0.6%)
Pulmonary infection	110 (14.2%)
Infectious pleural effusion	7 (0.9%)
Catheter-related sepsis	6 (0.8%)
Urinary infection	8 (1.0%)

A total of 207 pathogens were isolated from 156 patients and 180 infection sites. The specimens collected from corresponding infection sites included abdominal drainage fluids, bile, ascites, incision secretions, sputum, throat swabs, thoracic drainage fluid, catheter samples, and urine. Among the identified pathogens, gram-positive bacteria accounted for 39.6% (82 isolates), gram-negative bacteria accounted for 43.55% (90 isolates), and fungi accounted for 16.9% (35 isolates). Specifically, regarding gram-positive bacterial infections, *Staphylococcus aureus* constituted 3.9% (30 isolates), Coagulase-negative *staphylococci* (CNS) constituted 4.1% (32 isolates), *Enterococcus* constituted 2.2% (17 isolates), while others were present in smaller proportions as well. Among these strains of gram-positive bacteria that were identified, a total of 44 antibiotic-resistant strains were found, including 18 MRSA, 25 methicillin-resistant CNS (MRCNS), and 1 vancomycin-resistant *enterococcus* (VRE).

In terms of gram-negative bacterial infections, *Klebsiella pneumoniae* accounted for 3.1% (24 isolates), *Escherichia coli* accounted for 1.8% (14 isolates), *Enterobacter cloacae* accounted for 0.6% (5 isolates), *Acinetobacter baumannii* accounted for 4.3% (33 isolates), *Pseudomonas aeruginosa* accounted for 0.8% (6 isolates), and *Stenotrophomonas maltophilia* accounted for 0.5% (4 isolates). Other gram-negative bacteria of smaller proportions were also identified in the study population. Among these gram-negative bacteria, a total of 35 antibiotic-resistant strains were detected, including 8 CRE, 7 extended-spectrum beta-lactamases (ESBLs)-producing bacteria, 14 carbapenem-resistant *Acinetobacter baumannii* (CRAB), 2 carbapenem-resistant *Pseudomonas aeruginosa* (CRPA), and 4 intrinsically carbapenem-resistant *Stenotrophomonas maltophilia*. In addition to bacterial infections, 35 fungal infections were observed in this study, with *Candida albicans* accounting for the majority (32 isolates). A detailed summary of pathogens and their corresponding antibiotic resistance profiles can be found in Table 2.

**TABLE 2:** Summary of pathogens and antibiotic resistance

Pathogens	Pathogens (*n* = 207)	Antibiotic resistance
Gram-positive bacteria		
*Staphylococcus aureus (SA*)	30 (3.9%)	MRSA 18 (60.0%)
*Coagulase-negative staphylococci (CNS*)	32 (4.1%)	MRCNS 25 (78.1%)
*Enterococcus*	17 (2.2%)	VRE 1 (5.9%)
Others	3 (0.4%)	
Gram-negative bacteria		
*Klebsiella pneumoniae (KP*)	24 (3.1%)	CRE 7 (29.2%)
*Escherichia coli*	14 (1.8%)	CRE 1 (7.1%) ESBLs 5 (35.7%)
*Enterobacter cloacae*	5 (0.6%)	ESBLs 2 (40.0%)
*Acinetobacter baumannii (AB*)	33 (4.3%)	CRAB 14 (42.4%)
*Pseudomonas aeruginosa (PA*)	6 (0.8%)	CRPA 2 (33.3%)
*Stenotrophomonas maltophilia*	4 (0.5%)	Intrinsically CR
Others	4 (0.5%)	
Fungus		
*Candida albicans*	32 (4.1%)	
Others	3 (0.4%)	

### Comparisons between the infection group and the non-infection group

Subsequently, we conducted an analysis of recipient, donor, and surgery characteristics between patients with and without infection after LT (Table 3). The age of the infection group was significantly higher than that of the non-infection group (49.44 vs 46.71, *P* = 0.001). There was a significantly higher proportion of female patients in the infection group compared to the non-infection group (21.4% vs 14.6%, *P* = 0.049). Bilirubin levels before LT were significantly elevated in the infection group compared to the non-infection group (113.4 vs 83.9, *P* = 0.045). Furthermore, there were significant differences observed in terms of operation duration, mechanical ventilation duration, and ICU stay between the infection and non-infection groups (*P* = 0.003, *P* < 0.001, and *P* = 0.024, respectively). All other characteristics remained similar between both groups.

**TABLE 3:** Comparisons between infection group and non-infection group

	Infection (*n* = 156)	Non-infection (*n* = 620)	*P* value
Recipient			
Age (years)	49.44 + 9.20	46.71 + 10.23	0.001
Sex			0.049
Male	121 (78.6%)	526 (85.4%)	
Female	33 (21.4%)	90 (14.6%)	
Etiology			0.279
HBV	119 (77.3%)	510 (82.4%)	
HCV	2 (1.3%)	6 (1.0%)	
AIH	17 (11.0%)	47 (7.6%)	
Alcoholic hepatitis	7 (4.5%)	12 (1.9%)	
Congenital liver disease	0 (0%)	3 (0.5%)	
Wilson disease	3 (1.9%)	15 (2.4%)	
Drug-induced liver injury	0 (0%)	2 (0.3%)	
Iatrogenic liver injury	0 (0%)	8 (1.3%)	
Unknown	6 (3.9%)	16 (2.6%)	
Diabetes			0.260
Yes	14 (9.0%)	71 (12.2%)	
No	142 (91.0%)	510 (87.8%)	
Cirrhosis			0.893
Yes	126 (80.8%)	497 (80.3%)	
No	30 (19.2%)	122 (19.7%)	
Albumin (g/L)	34.82 + 6.91	33.86 + 6.39	0.105
Creatinine (μmol/L)	71.99 + 64.85	80.78 + 88.37	0.245
Bilirubin (μmol/L)	113.4 + 169.6	83.90 + 131.7	0.045
Ascites			0.395
No	76 (48.7%)	294 (47.4%)	
Mild to moderate	42 (26.9%)	198 (31.9%)	
Severe	38 (24.4%)	128 (20.6%)	
AFP > 400 ng/ml			0.075
Yes	15 (9.6%)	94 (15.2%)	
No	141 (90.4%)	526 (84.8%)	
Child-Pugh			0.488
A	54 (34.6%)	220 (35.5%)	
B	57 (36.5%)	249 (40.2%)	
C	45 (28.8%)	151 (24.4%)	
MELD	14.99 + 7.95	14.24 + 8.88	0.367
Donor			
Age (years)	30.52 + 9.29	31.03 + 10.78	0.766
Sex			0.349
Male	101 (98.1%)	455 (96.2%)	
Female	2 (1.9%)	18 (3.8%)	
Albumin (g/L)	35.04 + 6.66	33.69 + 9.04	0.605
Bilirubin (μmol/L)	22.30 + 19.16	15.65 + 12.24	0.103
ALT (U/L)	54.38 + 37.06	48.49 + 67.51	0.747
Surgery			
Warm ischemia time (min)	3.51 + 0.85	3.57 + 1.23	0.517
Cold ischemia time (h)	8.13 + 2.60	8.22 + 2.49	0.806
Time of anhepatic phase (min)	43.90 + 10.40	46.12 + 21.41	0.113
Time of operation (h)	7.00 + 1.76	6.52 + 1.76	0.003
Blood loss (mL)	2155 + 2461	1782 + 2163	0.178
Time of mechanical ventilation (h)	157.0 + 331.9	51.65 + 128.4	<0.001
Time in ICU after LT (h)	488.9 + 1582	183.5 + 295.7	0.024
Pre-LT immune checkpoint inhibitor	3 (1.9%)	10 (1.6%)	1.000
Post-LT without prednisone	52 (33.3%)	239 (38.5%)	0.267

### Comparisons between sepsis group and non-sepsis group

According to the sepsis-3 criteria, a total of 156 infected patients were categorized into either the sepsis or non-sepsis group. Subsequently, we conducted a comparative analysis of recipient, donor, and surgery characteristics between these two groups consisting of 62 septic patients and 94 non-septic patients (Table 4). Regarding the presence of infected pathogens, it was observed that drug-resistant bacteria accounted for 53.2% in the septic patient group compared to only 30.9% in the non-septic patient group (*P* = 0.005). Furthermore, there was a significant difference in mechanical ventilation duration between the sepsis and non-sepsis groups with values of 242.8 and 97.85, respectively (*P* = 0.020).

**TABLE 4:** Comparisons between sepsis group and non-sepsis group

	Sepsis (*n* = 62)	Non-sepsis (*n* = 94)	*P* value
Recipient			
Age (years)	49.02 ± 9.52	49.72 ± 9.01	0.640
Sex			0.655
Male	50 (80.6%)	73 (77.7%)	
Female	12 (19.4%)	21 (22.3%)	
Etiology			0.927
HBV	48 (78.7%)	71 (76.3%)	
HCV	0 (0%)	2 (2.2%)	
AIH	7 (11.5%)	10 (10.8%)	
Alcoholic hepatitis	2 (3.3%)	5 (5.4%)	
Wilson disease	1 (1.6%)	2 (2.2%)	
Unknown	3 (4.9%)	3 (3.2%)	
Diabetes			0.163
Yes	8 (12.9%)	6 (6.4%)	
No	54 (87.1%)	88 (93.6%)	
Cirrhosis			0.091
Yes	46 (74.2%)	80 (85.1%)	
No	16 (25.8%)	14 (14.9%)	
Albumin (g/L)	34.46 ± 6.67	35.05 ± 7.09	0.612
Creatinine (μmol/L)	79.56 ± 98.21	67.07 ± 26.06	0.243
Bilirubin (μmol/L)	146.04 ± 186.91	92.29 ± 154.81	0.064
Ascites			0.329
No	28 (45.2%)	48 (51.1%)	
Mild to moderate	15 (24.2%)	27 (28.7%)	
Severe	19 (30.6%)	19 (20.2%)	
AFP >400 ng/ml			0.564
Yes	7 (11.3%)	8 (8.5%)	
No	55 (88.7%)	86 (91.5%)	
Child-Pugh			0.086
A	18 (29.0%)	36 (38.3%)	
B	20 (32.3%)	37 (39.4%)	
C	24 (38.7%)	21 (22.3%)	
MELD	14.99 ± 7.95	14.24 ± 8.88	0.061
Drug-resistance bacteria			0.005
Yes	33 (53.2%)	29 (30.9%)	
No	29 (46.8%)	65 (69.1%)	
Donor			
Age (years)	28.91 ± 8.24	32.13 ± 10.16	0.245
Sex			1.000
Male	45 (97.8%)	56 (98.2%)	
Female	1 (2.2%)	1 (1.8%)	
Albumin (g/L)	34.22 ± 6.09	35.65 ± 7.41	0.706
Bilirubin (μmol/L)	28.70 ± 24.30	16.71 ± 12.30	0.240
ALT (U/L)	65.29 ± 46.01	44.84 ± 26.54	0.304
Surgery			
Warm ischemia time (min)	3.56 ± 0.88	3.46 ± 0.82	0.514
Cold ischemia time (h)	8.17 ± 2.61	8.10 ± 2.61	0.920
Time of anhepatic phase (min)	59.07 ± 11.14	58.77 ± 9.87	0.876
Time of operation (h)	7.19 ± 1.90	6.87 ± 1.66	0.264
Blood loss (mL)	3459 ± 2661	2964 ± 2327	0.317
Time of mechanical ventilation (h)	242.8 ± 421.5	97.85 ± 237.6	0.020
Time in ICU after LT (h)	625.6 ± 473.7	733.0 ± 2028	0.693
Pre-LT immune checkpoint inhibitor	1 (1.6%)	2 (2.1%)	1.000
Post-LT without prednisone	15 (24.2%)	37 (39.4%)	0.057

### Risk factors of infection and sepsis

The univariate analysis revealed that recipient age, sex, bilirubin levels, AFP levels, time of operation, duration of mechanical ventilation, and length of stay in the ICU were identified as potential variables associated with post-LT infection (*P* < 0.10). Additionally, recipient cirrhosis status, bilirubin levels, Child-Pugh grade, MELD score, drug-resistant bacterial infection, and duration of mechanical ventilation were found to be potential variables associated with post-LT sepsis (*P* < 0.10). These variables were subsequently dichotomized and subjected to multivariate analysis, according to our previous studies (2, 16).

Recipients aged >50 years (OR 1.70, *P* = 0.009), with mechanical ventilation duration >72 h (OR 3.04, *P* < 0.001), and ICU stay after LT >10 days (OR 3.00, *P* < 0.001) exhibited a significantly higher risk of post-LT infection. Among these infected recipients, those with mechanical ventilation duration >72 h (OR 2.57, *P* = 0.021), bilirubin levels >90 µmol/L (OR 3.46, *P* = 0.005), and drug-resistant bacterial infections (OR 2.35, *P* = 0.033) were more likely to develop sepsis (Table 5).

**TABLE 5:** Independent risk factors for infection and developing sepsis after LT

	Model I (*n* = 669)		Model II (*n* = 133)	
Age >50 years	1.70 (1.15–2.54)	0.009		
Time of mechanical ventilation >72 h	3.04 (1.94–4.75)	<0.001	2.57 (1.15–5.72)	0.021
Duration in ICU after LT >10 days	3.00 (1.76–5.14)	<0.001		
Bilirubin >90 µmol/L			3.46 (1.46–8.24)	0.005
Drug-resistance bacteria			2.35 (1.07–5.15)	0.033

## DISCUSSION

Infection and sepsis significantly impact the prognosis of LT patients, even in an era where 5-year survival rates are nearly 80% (2, 3, 17). Post-LT infections caused by antibiotic-resistant bacteria have become increasingly common, including the lethal carbapenem-resistant gram-negative bacteria. A decade ago, we previously reported on the spectrum of MDR gram-negative bacterial infections post-LT (4). However, most studies have focused only on specific infectious pathogens, and there is a lack of comprehensive analysis on serious post-LT infections such as sepsis, which plays a crucial role in determining prognosis. Therefore, this study aims to provide a comprehensive analysis of infection patterns and resistance profiles while simultaneously investigating risk factors for sepsis in LT patients. Furthermore, building upon our previous study conducted from 2007 to 2010, we aim to demonstrate the shifting pattern of bacterial infections in recent decades within the field of LT.

**Fig 1:**
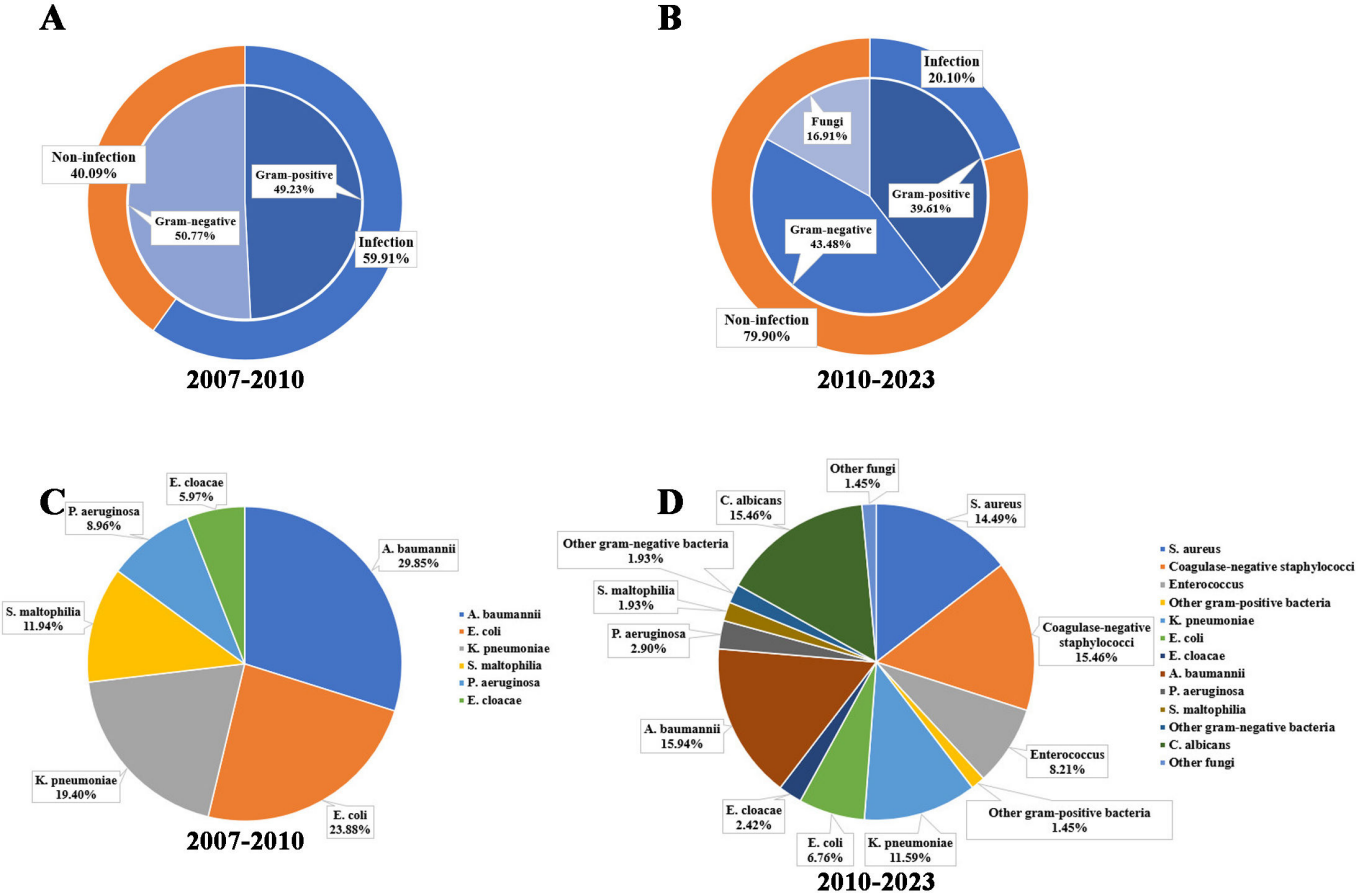
Infection and pathogen spectrum during the periods of 2007–2010 and 2010–2023. (**A**) Bacterial infection spectrum during the period of 2007–2010. (**B**) Infection spectrum during the period of 2010–2023. (**C**) MDR gram-negative bacteria spectrum during the period of 2007–2010. (**D**) Pathogen spectrum during the period of 2010–2023.

In our previous study, the incidence of bacterial infection was as high as 59.9%. Specifically, 29.5% were attributed to gram-positive bacteria and 30.4% to gram-negative bacteria (Fig. 1A) (4). In this large cohort study, a total of 20.1% of patients were infected by either bacteria or fungi (Fig. 1B). Although there has been a remarkable decrease in the overall bacterial infection rate over recent years, the ratio between gram-negative and gram-positive bacteria remains nearly unchanged at approximately 1:1 (Fig. 1A and B). This significant improvement can be attributed to several key factors, including advancements in infection prevention protocols, refinements in immunosuppressive therapies, improvements in surgical techniques, reduced intubation times, and enhanced peri-operative management practices. The pathogen spectrums of 2007–2010 (MDR gram-negative bacteria) and 2010–2023 were shown in Fig. 1C and D. Among specific gram-negative pathogens identified, the top three were *Acinetobacter baumannii* (36.7%), *Klebsiella pneumoniae* (26.7%), and *Escherichia coli* (15.6%), which aligns with previous reports from years ago (4). However, it is noteworthy that both *Acinetobacter baumannii* and *Klebsiella pneumoniae* have shown an increase of approximately 6%–7%, while *Escherichia coli* has decreased by 8.3%. Furthermore, carbapenem resistance was observed in up to 31.1% of gram-negative bacteria including *Acinetobacter baumannii* (42.4%), *Klebsiella pneumoniae* (29.2%), *Escherichia coli* (7.1%), *Pseudomonas aeruginosa* (33.3%) and *Stenotrophomonas maltophilia* (100%).

Without a doubt, bacterial infection is the most prevalent post-LT infection. The incidence of bacterial infection varies among different transplantation centers and eras. Previous studies have reported incidences of post-LT infection ranging from 18.4% (18), 26.6% (19), 44% (20), and 68.6% (21) in these centers, respectively. In our study, the infection rate was determined to be 20.1%. Notably, the spectrum of infections was significantly associated with geographical regions and environmental factors, while perioperative management further influenced post-LT infections’ occurrence and characteristics. Therefore, studying the spectrum of post-LT infections within a single center holds great significance. This large-scale study conducted at a single center in East China successfully revealed the regional spectrum of post-LT infections.

Infections caused by MDR bacteria, particularly carbapenem-resistant gram-negative bacteria, have emerged as a significant public health challenge due to limited antibiotic options and high case-fatality rates (22). The Centers for Disease Control and Prevention (CDC) has identified CRE and CRAB as two of the five urgent threats to public health. Additionally, the World Health Organization (WHO) has classified CRAB, CRPA, and CRE as priority 1 (critical) pathogens requiring research, discovery, and development of new antibiotics (23). According to the most recent data from CHINET (https://www.chinets.com/Data/AntibioticDrugFast), approximately 20% of *Pseudomonas aeruginosa* isolates are carbapenem-resistant along with 78% of *Acinetobacter baumannii* isolates and 30% of *Klebsiella pneumoniae* isolates. Overall, our study found that 31.1% of GNBs were resistant to carbapenems. Furthermore, we observed common resistance among post-LT infections caused by MRSA, MRCNS, VRE, and ESBLs. Importantly, LT patients infected with these drug-resistant pathogens had a higher likelihood of developing sepsis (OR 2.351; *P* = 0.033). Previous studies have reported that infection with MDR pathogens significantly increases the risk of inappropriate empiric therapy administration and mortality (24–26). Based on our findings presented in this study, we propose that the association between infection with MDR pathogens and increased mortality may be attributed to a higher incidence rate of subsequent sepsis.

Other independent risk factors for infection and sepsis in our study include advanced age, prolonged mechanical ventilation, extended ICU stay, and elevated bilirubin levels. Advanced age has been widely recognized as a well-known risk factor for infection and has been consistently identified in previous studies (9, 27). In our previous study (4), we also established prolonged mechanical ventilation as an independent risk factor for both GNB infection and MDR organisms. Here, we further demonstrate that prolonged mechanical ventilation is associated with an increased risk of sepsis development among recipients. LT patients are particularly susceptible to sepsis due to various factors such as cirrhosis-associated immune dysfunction, frequent hospital admissions, multiple antibiotic courses, lengthy stays in the ICU, and immunosuppressive therapies (2, 28). Yoshizumi et al. reported that a MELD >15 was an independent risk factor for bacterial sepsis development (29). Regarding the MELD score, our findings indicate that bilirubin levels exceeding 90 µmol/L constitute a significant risk factor for the development of sepsis. Furthermore, other studies have also reported an association between elevated bilirubin levels, sepsis occurrence, and subsequent outcomes (30, 31). Notably, bilirubin >90 µmol/L serves as a more specific and modifiable indicator of liver function impairment. This finding suggests the importance of implementing jaundice-reducing treatments prior to LT to mitigate the occurrence of post-LT sepsis. In our previous study, we identified post-LT without the administration of prednisone as an independent protective factor against GNB infection. In contrast, in this study, the use of post-LT prednisone showed only a non-significant trend. This discrepancy may be attributed to a significantly lower incidence of infection or advancements in perioperative management.

There are several limitations inherent to this retrospective study. Firstly, the retrospective nature of the study inherently imposes certain limitations. This study is a continuous cohort study spanning 14 years. It is important to acknowledge that such a long-term study may introduce potential biases, such as loss to follow-up, time effects, and confounding factors. However, our center has implemented robust follow-up procedures to minimize these issues. Secondly, it is important to note that this study was conducted at a single center. While conducting a multi-center study on infection patterns may be impractical, it would be valuable to validate and compare these findings using data from other transplantation centers. Finally, the correlation between immune checkpoint inhibitors and infection after LT remains uncertain and warrants further investigation; however, it should be noted that this study has a limited sample size.

### Conclusions

Bacterial infection was predominant in post-LT infections. Currently, the proportion of gram-negative bacterial infections after LT is comparable to that of gram-positive bacterial infections. More than 45% of bacterial infections were caused by drug-resistant pathogens, with over 30% of gram-negative bacteria exhibiting carbapenem resistance. Recipients aged >50 years, those requiring mechanical ventilation for >72 h, and individuals with an ICU stay >10 days after LT were significantly more susceptible to post-LT infections. Among infected patients, those who required mechanical ventilation for >72 h, had bilirubin levels >90 µmol/L, and experienced drug-resistant bacterial infections were at a higher risk of developing sepsis. Based on these factors, strategies aimed at reducing the duration of mechanical ventilation and ICU stay may help decrease the incidence of post-LT infections. Additionally, implementing pre-LT treatments targeting jaundice reduction could potentially aid in preventing post-LT sepsis.

## Data Availability

The data that support the findings of this study are available from the corresponding author upon reasonable request. The data sets used and analyzed during the current study are available from the corresponding author on reasonable request.

## SUPPLEMENTAL MATERIAL

Table S1 spectrum.00711-25-s0001.docx Summary of recipient, donor, and surgery characteristics.

ASM does not own the copyrights to Supplemental Material that may be linked to, or accessed through, an article. The authors have granted ASM a non-exclusive, world-wide license to publish the Supplemental Material files. Please contact the corresponding author directly for reuse.
