# Supplementary material for: Epidemiology, antimicrobial resistance, and risk factors of infection among liver transplant patients: a retrospective study 2010–2023
Source: Microbiol Spectr. 2025 Jun 10;13(7):e00711-25. doi: 10.1128/spectrum.00711-25 (PMC12210900; doi:10.1128/spectrum.00711-25)
Supplement: Table S1 — Summary of recipient, donor, and surgery characteristics. [file spectrum.00711-25-s0001.docx]

**Supplementary Table. 1 Summary of recipient, donor and surgery characteristics**

| N=776 | |
| --- | --- |
| **Recipient** | |
| Age | 47 [41 - 54] |
| Sex  Male  Female | 647 (83.4%)  129 (16.6%) |
| Etiology  HBV  HCV  AIH  Alcoholic hepatitis  Congenital liver disease  Wilson disease  Drug-induced liver injury  Iatrogenic liver injury  Unknown | 629 (80.3%)  8 (1.1%)  64 (8.3%)  19 (2.5%)  30 (3.6%)  18 (2.3%)  3 (0.4%)  8 (1%)  22 (2.8%) |
| Diabetes | 85 (11%) |
| Cirrhosis | 597 (76.9%) |
| Albumin (g/L) | 34.05 + 6.51 |
| Creatinine (μmol/L) | 79.02 + 84.23 |
| Bilirubin (μmol/L) | 89.81 + 140.5 |
| Ascites  No  Mild to moderate  Severe | 370 (47.7%)  240 (30.9%)  166 (21.4%) |
| AFP > 400 ng/mL  Yes  No | 109 (14.0%)  667 (86.0%) |
| Child-Pugh  A  B  C | 274 (35.3%)  306 (39.4%)  196 (25.3%) |
| MELD | 14.43 + 8.64 |
| **Donor** |  |
| Age | 28 [25 - 36] |
| Sex  Male  Female | 749 (96.5%)  27 (3.5%) |
| Albumin (g/L) | 33.97 + 8.56 |
| Bilirubin (μmol/L) | 17.02 + 14.04 |
| AST (U/L) | 48.17 + 40.95 |
| ALT (U/L) | 49.72 + 62.22 |
| **Surgery** |  |
| Warm ischemia time (minutes) | 3.56 + 1.16 |
| Cold ischemia time (hours) | 8.19 + 2.52 |
| Time of anthepatic phase (minutes) | 45.42 + 19.79 |
| Time of operation (hours) | 6.62 + 1.77 |
| Blood loss (mL) | 1895 + 2260 |
| Time of mechanical ventilation (hours) | 72.7 + 192.0 |
| Time in ICU after LT (hours) | 446.3 + 772.1 |
| Pre-LT immune checkpoint inhibitor | 13 (1.7%) |
| Post-LT without prednisone | 291 (37.5) |

AFP, alpha fetoprotein; AIH, autoimmune hepatitis; ALT, alanine transaminase; AST, aspartate transaminase; HBV, hepatitis B virus; HCV, hepatitis C virus; ICU, intensive care unit; LT, liver transplantation; MELD, model for end-stage liver disease.
